# Bilirubin Increases Insulin Sensitivity by Regulating Cholesterol Metabolism, Adipokines and PPARγ Levels

**DOI:** 10.1038/srep09886

**Published:** 2015-05-28

**Authors:** Jinfeng Liu, Huansheng Dong, Yong Zhang, Mingjun Cao, Lili Song, Qingjie Pan, Andrew Bulmer, David B. Adams, Xiao Dong, Hongjun Wang

**Affiliations:** 1 College of Animal Science and Veterinary Medicine, Qingdao, P.R. China; 2 Department of Surgery, Medical University of South Carolina, Charleston, SC 29425, USA; 3 Colleges of Life Sciences, Qingdao Agricultural University, Qingdao, P.R. China; 4 School of Medical Science, Griffith University, Australia

## Abstract

Obesity can cause insulin resistance and type 2 diabetes. Moderate elevations in bilirubin levels have anti-diabetic effects. This study is aimed at determining the mechanisms by which bilirubin treatment reduces obesity and insulin resistance in a diet-induced obesity (DIO) mouse model. DIO mice were treated with bilirubin or vehicle for 14 days. Body weights, plasma glucose, and insulin tolerance tests were performed prior to, immediately, and 7 weeks post-treatment. Serum lipid, leptin, adiponectin, insulin, total and direct bilirubin levels were measured. Expression of factors involved in adipose metabolism including sterol regulatory element-binding protein (SREBP-1), insulin receptor (IR), and PPAR**γ** in liver were measured by RT-PCR and Western blot. Compared to controls, bilirubin-treated mice exhibited reductions in body weight, blood glucose levels, total cholesterol (TC), leptin, total and direct bilirubin, and increases in adiponectin and expression of SREBP-1, IR, and PPARγ mRNA. The improved metabolic control achieved by bilirubin-treated mice was persistent: at two months after treatment termination, bilirubin-treated DIO mice remained insulin sensitive with lower leptin and higher adiponectin levels, together with increased PPAR**γ** expression. These results indicate that bilirubin regulates cholesterol metabolism, adipokines and PPAR**γ** levels, which likely contribute to increased insulin sensitivity and glucose tolerance in DIO mice.

Obesity has reached epidemic proportions globally, and at least 2.8 million people die each year as a result of being overweight or obese (WHO). Obesity also represents the most important risk factor for insulin resistance, cardiovascular diseases, and type 2 diabetes (T2D)^1^. Patients with T2D are commonly hyperglycemic, and suffer complications such as hypertension, stroke, atherosclerosis, and cancer^2^. Strategies that reduce obesity promise to reduce mortality and improve quality of life of affected individuals.

Adipose tissue plays a critical role in glucose homeostasis and lipid metabolism^3^,^4^ via production of various proteins known as adipokines or adipocytokines including tumor necrosis factor-α (TNF-α), interleukin-6, monocyte chemoattractant protein 1, leptin, and adiponectin^5^,^6^,^7^. Adipokines act as metabolic switches, connecting the body’s nutritional status to other energy consuming functions^8^. Dysregulation of adipokines leads to obesity related diseases^9^. Among known adipokines, leptin levels positively correspond with energy stored in the fat mass, and elevated leptin levels correlate with increased adiposity and inflammation resulting from an increased release of cytokines, such as TNF-α^10^. In contrast, adiponectin acts as an insulin-sensitizing agent and enhances fatty acid oxidation, liver insulin action, and glucose uptake^5^,^11^,^12^. Adiponectin deficient mice exhibit elevated plasma glucose and insulin levels after being fed a high-fat diet, but overexpression of adiponectin in these mice by adenoviral infection restores insulin sensitivity^13^. In addition, adiponectin also has anti-inflammatory and anti-atherogenic effects resulting from its capacity to suppress TNF-α-mediated NF-κB activation in endothelial cells^14^. Circulating adiponectin levels are increased following weight loss^15^, by induction of heme oxygenase-1 (HO-1)^16^, and by treatment with PPARγ ligands, including thiazolidinediones^17^,^18^.

HO-1 is a rate-limiting enzyme that degrades heme generating equal molar amounts of biliverdin, carbon monoxide, and ferrous iron^19^. Biliverdin can be rapidly converted into bilirubin by biliverdin reductase, and iron can up-regulate ferritin. HO-1 has been shown to play critical roles in reversing insulin resistance. For example, HO-1 induction sensitizes the insulin signaling pathway in Zucker rats^20^ and in leptin deficient (ob/ob) mice, in leptin-receptor deficient (db/db) mice, and in diet induced obese (DIO) mice^16^,^21^,^22^,^23^,^24^,^25^,^26^,^27^. Bilirubin mimics the effect of HO-1 induction and improves insulin sensitivity in obese mouse models^28^. Bilirubin is a strong anti-oxidant with anti-inflammatory and immune regulatory properties^29^. Although abnormally elevated bilirubin has been used as an indicator for hepatitis, hemolytic anemia, and cholestasis, mildly elevated unconjugated bilirubin in patients with Gilbert’s syndrome (1.1 mg/dl to 2.7 mg/dl) is associated with protection from coronary atherosclerosis, cardiovascular disease, as well as other diseases^30^,^31^,^32^,^33^. The positive association between unconjugated bilirubin and free plasma heme, iron, and carboxyl hemoglobin in these patients suggests a positive feedback loop in which HO-1 expression is induced by unconjugated bilirubin^34^.

We have shown that systemic administration of bilirubin increases insulin sensitivity via suppressing ER stress and inflammation in DIO mice^35^. We have now extended these observations and have investigated whether bilirubin regulates lipid metabolism as seen in patients with Gilbert’s disease^34^ and whether bilirubin regulates serum adipokine levels in DIO mice. We also assessed longer-term effects of bilirubin treatment on lipid metabolism and adipokine levels 7 weeks after completion of bilirubin treatment.

## Results

### Bilirubin administration reduces body weight and ameliorates insulin resistance in DIO mice

**Figure 1:**
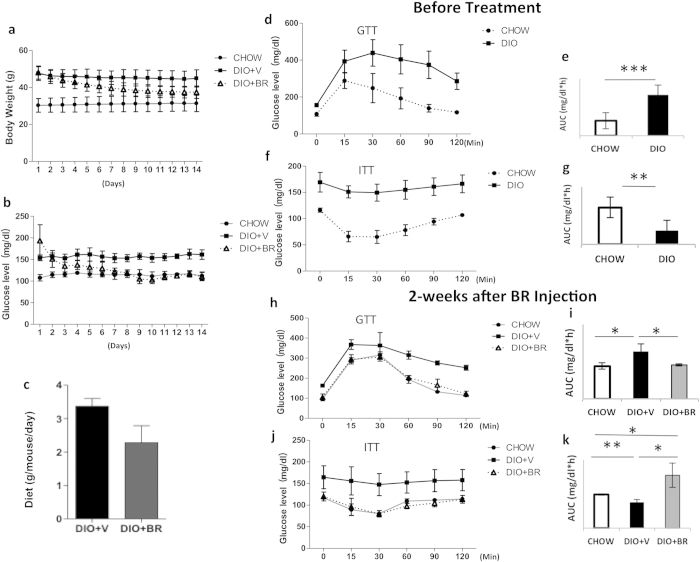
Administration of bilirubin reduces body weight and increases insulin sensitivity in DIO mice. (**a**) Changes in body weights of DIO mice treated with bilirubin (DIO + BR) or vehicle (DIO + V) compared to control mice fed standard diet (CHOW). (**b**) Daily non-fasting blood glucose levels in DIO + BR, DIO + V, and CHOW mice during bilirubin treatment. (**c**) Average food intake per mouse per 24-h period in DIO mice receiving BR or vehicle. (**d**) Intraperitoneal glucose tolerance test (GTT) of DIO mice and CHOW controls before bilirubin treatment; (**e**) area under the curve of GTT. (**f**) Insulin tolerance test (ITT) of DIO mice and CHOW mice before bilirubin treatment; (**g**) reverse area under the baseline above curve. (**h**) GTT of DIO + BR, DIO + V, and CHOW mice 14 days after the first bilirubin injection; (**i**) area under the curve. (**j**) ITT of DIO + BR, DIO + V, and CHOW mice at 14 days after the first bilirubin injection; (**k**) reverse area under the baseline above curve. At least 6-8 mice were included in each group; **p < 0.01, *p < 0.05, *Student’s t* test. DIO + BR: DIO mice treated with bilirubin; DIO + V: DIO mice treated with vehicle; CHOW: mice fed standard diet.

The effects of bilirubin administration on body weight, blood glucose level, and insulin sensitivity in DIO mice were determined. Animals were treated with bilirubin (20 μmol/kg) intraperitoneally twice per day for 14 days. This dose and dosing schedule has shown efficacy and was well tolerated in previously published islet transplantation and obese mouse models^35^,^36^. Feeding a high fat diet (Table 1) to C57BL/6 mice for 24 weeks resulted in significant increases in body weight (47.5 ± 3.7 g in DIO vs. 30.29 ± 3.5 in mice fed standard diet (CHOW), Fig. 1a). Bilirubin administration (DIO + BR) significantly reduced body weights in DIO mice (Fig. 1a). Blood glucose levels were reduced in bilirubin treated mice from 193.5 ± 37.1 mg/dl to 112.2 ± 8.9 mg/dl (Fig. 1b). Bilirubin treated mice ate less than DIO controls; however, this difference was not significant (p = 0.06, Fig. 1c). Bilirubin treatment resulted in improved glucose uptake and insulin utilization as evidenced by reduced blood glucose levels as well as reduced areas under the curve (GTT, Fig. 1h,j) or reverse areas above the curve (ITT, Fig. 1i,k), compared to GTT (Fig. 1d,e) and ITT (Fig. 1f,g) results before starting treatment. These data confirmed that bilirubin treatment reduced obesity and blood glucose levels and improved glucose tolerance and insulin sensitivity in DIO mice.

### Bilirubin administration reduces liver and fat weights by reducing adiposity in those tissues

**Figure 2:**
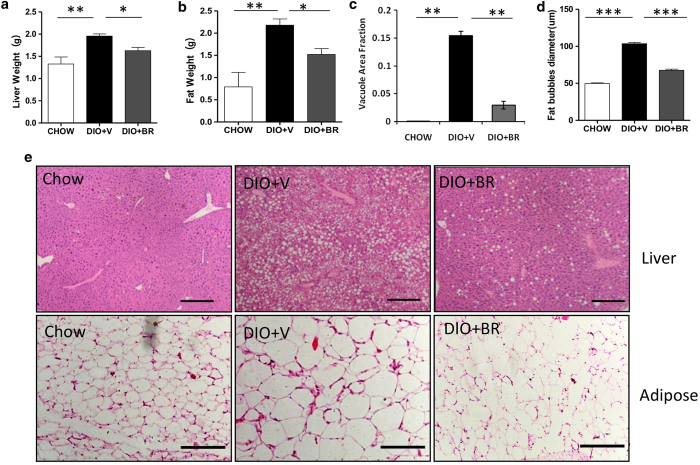
Bilirubin treatment reduces liver and fat weights by reducing adiposity 14 days after initiating bilirubin treatment. (**a**) Liver weights of DIO + BR, DIO + V, and CHOW mice. (**b**) Epididymal fat weights of DIO + BR, DIO + V, and CHOW mice. (**c**) Vacuole area fractions in livers of DIO + BR, DIO + V, or CHOW mice. (**d**) Fat bubble diameters (μm) of epididymal adipocytes of DIO + BR, DIO + V, or CHOW. (**e**) Representative micrographs of H&E staining of liver and adipose tissue sections from DIO + BR, DIO + V, and CHOW mice. Scale bar = 200 μm. At least 3 mice were analyzed in each group; *p < 0.05, *Student’s t* test. Data are mean ± standard deviation.

To determine the effects of bilirubin on adiposity, liver and epididymal fat tissue were obtained from half of the mice in each treatment arm (n = 3-4) and were sacrificed after 14-days of bilirubin treatment. Mice in the bilirubin group had significantly reduced liver and epididymal fat weights (Fig. 2). Using hematoxylin and eosin (H&E) staining, we observed that treatment with bilirubin improved hepatic steatosis in DIO mice (Fig. 2c,e, upper panel) and reduced the average size of adipocytes in epididymal fat (Fig. 2d,e, lower panel).

### Bilirubin therapy alters cholesterol and adipokine levels in DIO mice

**Figure 3:**
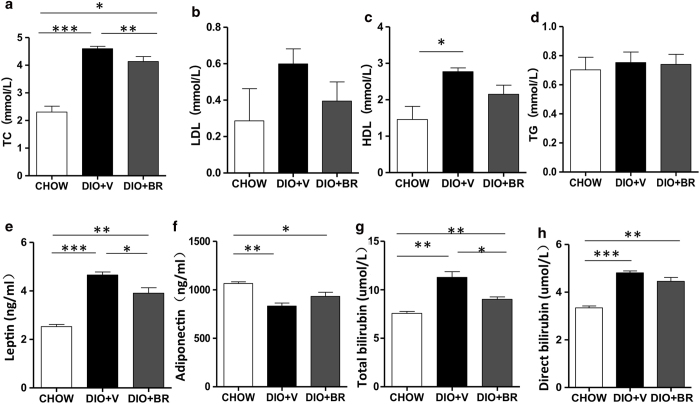
Bilirubin therapy alters cholesterol and adipokine levels in DIO mice at 14 days post treatment. Serum levels of TC (**a**), LDL (**b**), HDL (**c**), TG (**d**), leptin (**e**), adiponectin (**f**), total bilirubin (**g**), and direct bilirubin (**h**) in DIO + BR, DIO + V, or CHOW mice were measured in serum immediately after completing bilirubin treatment. Each group contained 3-4 mice; Data are mean ± standard deviation; ***p < 0.001, **p < 0.01, and *p < 0.05, *Student’s t* test.

We measured TC, LDL, HDL, and TG levels in DIO mice treated with bilirubin or vehicle and in CHOW controls at 14 days post treatment. The data indicated that DIO mice had elevated TC, LDL, and HDL levels compared to CHOW controls (Fig. 3a–c). Bilirubin treatment significantly reduced TC levels in DIO mice (p < 0.01). Both LDL and HDL were also lower in bilirubin-treated DIO mice compared to vehicle-treated controls, although the differences were not significant. No changes in TG levels were observed among the groups (Fig. 3d). We evaluated whether bilirubin reversed changes in leptin and adiponectin levels caused by high fat diet in DIO mice. Bilirubin treatment significantly reduced leptin and slightly increased adiponectin levels 14 days post treatment (Fig. 3e,f), which supports the hypothesis that the effects of bilirubin on obesity and insulin resistance are, at least in part, mediated by leptin and adiponectin. Total serum bilirubin levels and direct bilirubin levels were also reduced in bilirubin-treated mice compared to DIO controls (Fig. 3g,h). Serum insulin levels were slightly reduced in bilirubin treated mice compared to DIO controls, but the differences were not significant (*data not shown*).

### Bilirubin regulates the expression of genes related to obesity and insulin resistance in liver

**Figure 4:**
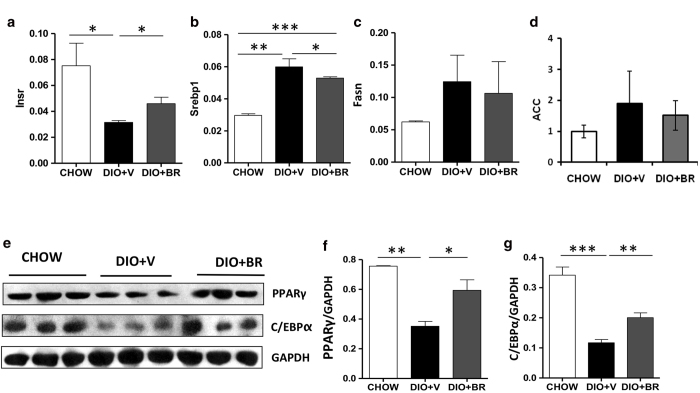
Bilirubin promotes expression of genes related to obesity and insulin resistance in liver 14 days post treatment. Relative mRNA expression of insulin receptor (Insr) (**a**), SREBP-1 (**b**), Fasn (**c**) and ACC (**d**) in livers of DIO + BR, DIO + V, and CHOW mice as measured by RT-PCR analysis. Values represent relative expression of target gene compared to expression of endogenous control. (**e**) Expression of PPARγ and C/EBPα protein determined in liver from DIO + BR, DIO + V, and CHOW mice by Western blot analysis. Gels had been run under same experimental conditions and cropped blots are shown. (**f**) Relative protein expression of PPARγ and (**g**) C/EBPα relative to glyceraldehyde 3-phosphate dehydrogenase (GAPDH) control (NIH ImageJ software). Samples from 3 individual mice were analyzed. Data are mean ± standard deviation; ***p < 0.001, **p < 0.01 and *p < 0.05, *Student’s t* test.

To examine the effect of bilirubin on hepatic lipid metabolism, we analyzed hepatic gene expression in DIO mice treated with vehicle or bilirubin and in CHOW controls. Bilirubin treatment reversed the decrease in insulin receptor (Insr) expression in liver caused by high fat diet (Fig. 4a). Moreover, bilirubin treatment significantly reduced expression of SREBP-1, a gene required for *de novo* lipogenesis (Fig. 4b). Expression levels of Fasn and acetyl-coA carboxylase (ACC) were not significantly different between DIO mice treated with bilirubin or vehicle (Fig. 4c,d). No differences in expression levels of the leptin receptor were observed among DIO mice treated with vehicle or bilirubin or CHOW controls (data not shown). Expression of PPARγ and CCAAT-enhancer-binding proteins (C/EBPs) were significantly reduced in DIO controls compared to CHOW but bilirubin treatment restored PPARγ levels and partially restored C/EBP levels (Fig. 4e–g), suggesting a possible role of PPARγ in the therapeutic effects of bilirubin.

### The insulin sensitizing effects of bilirubin persist after completion of bilirubin treatment

**Figure 5:**
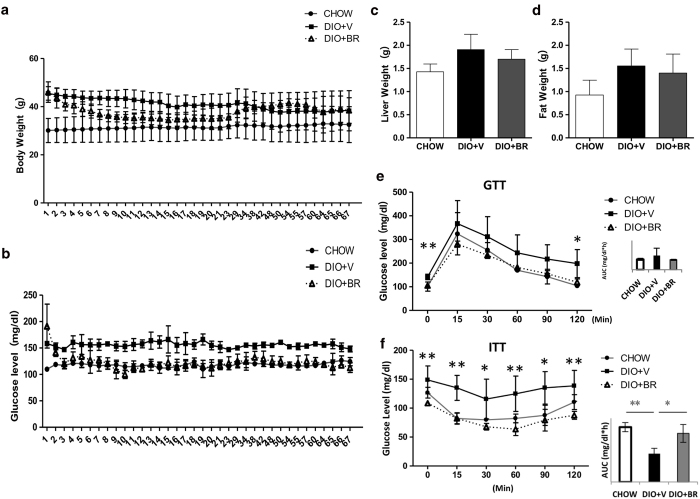
The insulin sensitizing effects of bilirubin persists 7 weeks after completion of bilirubin treatment. (**a**) Body weight of DIO + BR, DIO + V, and CHOW mice during the 9-week experiment. (**b**) Serum glucose levels in DIO + BR, DIO + V, and CHOW mice during the 9-week experiment. Liver (**c**) and epididymal fat weights (**d**) measured 7 weeks after treatment completion. (**e**) (left) GTT of DIO mice measured 7 weeks after treatment completion; (right) values of area under the curve above baseline; (**f**) (left) ITT of DIO mice measured 7 weeks after treatment completion; (right) values of reverse area above the curve under baseline. At least 3 mice were included in each group. Data are mean ± standard deviation; ***p < 0.001, **p < 0.01, *p < 0.05, *Student’s t* test.

**Figure 6:**
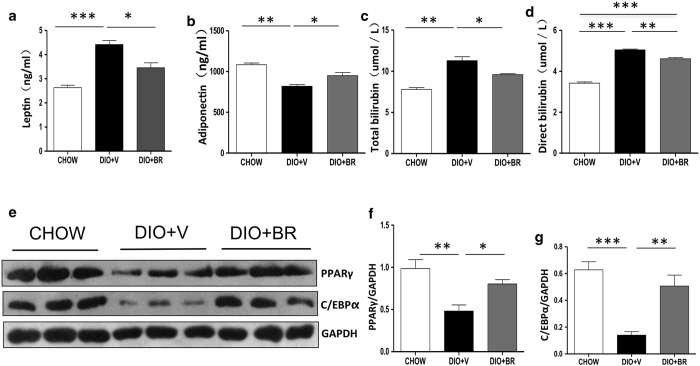
Gene expression in liver 7 weeks after completion of bilirubin treatment. Serum leptin (**a**), adiponectin (**b**), total bilirubin (**c**), and direct bilirubin (**d**) levels were measured in liver 7 weeks after completing bilirubin injection in DIO + BR, DIO + V, and CHOW mice. (**e**) Expression of PPARγ and C/EBPα protein determined in liver from DIO + BR, DIO + V, and CHOW mice by Western blot. (**f**) Relative protein expression of PPARγ and (**g**) C/EBPα relative to glyceraldehyde 3-phosphate dehydrogenase (GAPDH) control. Samples from 3 individual mice were analyzed. Data are mean ± standard deviation; ***p < 0.001, **p < 0.01 and *p < 0.05, *Student’s t* test.

To determine whether the therapeutic effect of bilirubin on body weight and insulin sensitivity persists after treatment termination, DIO mice were treated with bilirubin or vehicle for 14 days (n = 3–4 per group) and were maintained of the high fat diet for a further 7 weeks following treatment. Body weight, glucose tolerance, insulin sensitivity, blood chemistry, and gene expression in liver were measured. Body weights in bilirubin-treated DIO mice remained reduced for a further two weeks and then slowly returned to a level comparable to DIO controls (Fig. 5a). Liver and fat weights showed trends similar to the total body-weight differences observed after discontinuing bilirubin treatment (Fig. 5c,d). The results of this experiment indicate that the effects of bilirubin on body weight are temporary. In contrast, bilirubin-treated mice maintained their insulin sensitivity and glucose tolerance, as manifested by their normoglycemia (Fig. 5b) and their lower blood glucose levels following GTT and ITT tests (Fig. 5e,f), 7 weeks after discontinuing bilirubin treatment. There were no differences in food intake between groups throughout the experiment (data not shown). At 7 weeks post treatment termination, mice treated with bilirubin retained slightly lower serum cholesterol (2.8 ± 1.0 mmol/L vs. 3.9 ± 1.7 mmol/L, P = 0.19), TG (0.7 ± 0.2 mmol/L vs. 0.9 ± 0.3 mmol/L, P = 0.14), leptin (3.5 ± 0.4 ng/ml vs. 4.4 ± 0.3 ng/ml, P = 0.01), total bilirubin (9.6 ± 0.2 μmol/L vs. 11.3 ± 0.28 μmol/L, P < 0.05), direct bilirubin (4.6 ± 0.1 μmol/L vs. 5.4 ± 0. 06 μmol/L, P < 0.01), and higher adiponectin (949.5 ng/ml ± 64.2 vs. 819.0 ± 34.2 ng/ml, p = 0.02) levels compared to DIO controls (Fig. 6.a–d). Expression of insulin receptor remained slightly higher compared to vehicle-treated controls (data not shown). Expression of PPARγ and C/EBPs remained significantly higher in bilirubin treated DIO mice compared to vehicle treated DIO mice (Fig. 6e–g), suggesting that the insulin-sensitizing effect of bilirubin is at least in part mediated by PPARγ. Taken together, these data suggest that short-term bilirubin treatment temporarily affects body weight but its insulin-sensitizing effects persist long after treatment ends.

## Discussion

We recently identified a novel function for bilirubin as an insulin sensitizer that reduces obesity and hyperglycemia in obese mouse models^35^. The current study assessed the mechanisms of this protection. We first confirmed that a 14-day administration of bilirubin reduced body weight and increased insulin sensitivity in DIO mice without detectable side effects. The action of bilirubin was paralleled by a decrease in liver and fat weights, by decreases in serum total cholesterol, insulin, and leptin levels, and by increases in adiponectin levels. Bilirubin treatment reduced expression of SREBP-1, a gene critical for fatty acid synthesis, and restored expression in liver of insulin receptor, PPAR**γ,** and C/EBPα. Although bilirubin-treated mice gradually regained body weight after treatment termination, they remained sensitive to insulin stimulation 7 weeks post-treatment, and serum TC, leptin, and adiponectin levels and insulin receptor expression remained comparable to levels immediately after bilirubin treatment, and similar to levels in lean controls.

The most dramatic finding of this study is that treatment with bilirubin reduced total cholesterol levels in DIO mice. The reduction may be due to changes in both HDL and LDL levels, although reductions of these elements did not reach statistical significance. These findings are in agreement with observations in individuals with Gilbert’s syndrome in which mildly elevated bilirubin levels are associated with reduced total cholesterol, LDL, and triglyceride concentrations, and are associated with reduced pro-inflammatory status and reduced exposure to oxidative conditions^31^,^34^,^37^,^38^,^39^. Although we didn’t observe a change in triglyceride concentrations in bilirubin-treated mice 14 days after bilirubin treatment, a reduction was observed 7 weeks post treatment, indicating that the impact of bilirubin on triglyceride levels may be delayed. Our study provided evidence that exogenously administered bilirubin is associated with an altered lipid profile in obese mice.

We found that bilirubin administration significantly reduced leptin levels in DIO mice. Food intake by bilirubin-treated DIO mice did not appear to be a factor during bilirubin treatment, and no difference in food intake was observed after treatment ended. The reduction in leptin levels in bilirubin treated mice may be caused by a reduction in adipose tissue as supported by decreased expression of SREBP-1. Two other observations are relevant: leptin is known to augment inflammation through regulation of TNF-α^40^, and bilirubin treatment reduces TNF-α expression in DIO mice^35^. Therefore, it is reasonable to postulate that the effects of bilirubin on obesity are a consequence of altered lipid synthesis, reduction of leptin levels, and decreased TNF-α production. Of clinical relevance, bilirubin should be examined as a potential treatment to reduce lipid synthesis, as suggested by human studies^34^.

We found that bilirubin treatment resulted in increased PPARγ expression in liver. PPARγ is thought to be a “master” gene of adipocyte biology and differentiation^17^, and various PPARγ agonists including thiazolidinediones have been used as insulin sensitizers for the treatment of type 2 diabetes. Thiazolidinediones stimulate insulin sensitivity via upregulation of adiponectin, a critical adipokine that enhances fatty acid oxidation, liver insulin action, and glucose uptake 5,11,12. We observed increased adiponectin levels immediately after bilirubin treatment and 7 weeks after bilirubin treatment. HO-1, another PPARγ agonist, has been shown to increase insulin sensitivity through PPARγ upregulation and adiponectin production^5^. Bilirubin is known to upregulate HO-1 in humans^41^. We observed increased HO-1 induction in our bilirubin treated mice (Kim. *et al*. unpublished data). Therefore, it is possible that bilirubin exerts its protective effect on insulin sensitivity through upregulation of HO-1 and PPARγ. Of note, PPARγ levels were reported to be increased in mice fed with high fat diet for 2 weeks^42^. In contrast, we observed a decrease in PPARγ expression in vehicle-treated DIO mice. This disparity might be caused by the length of high fat-diet feeding since we fed our mice for 24 weeks to achieve obesity and insulin resistance. In addition, there are three PPARγ isotypes, and PPARγ2 has been shown to be required for adiponectin expression. The PPARγ antibody used in this study recognizes all three isotypes (PPARγ1, 2, and 3). Therefore, we did not distinguish specific isotypes of PPARγ that were upregulated by bilirubin treatment. Nevertheless, we did observe increased C/EBPα expression in mice treated with bilirubin.

As also reported by other investigators^43^,^44^, we found that DIO mice exhibited higher total and direct bilirubin levels than did Chow controls or DIO mice treated with exogenous bilirubin. The increased bilirubin in DIO animals may reflect blockage of bilirubin excretion due to impaired liver function in these mice. One possible explanation for the reduction in bilirubin levels in bilirubin treated DIO mice is that the reduction may be a consequence of the ability of exogenous bilirubin to regulate P450. P450s are a superfamily of heme-containing monooxygenase enzymes that metabolize a diverse range of compounds of both endogenous and exogenous origin. Studies have shown that the two different isoforms of P450, CTP1A1 and CYP1A2 mediate an alternate route for bilirubin degradation^45^,^46^,^47^. In addition, bilirubin can directly regulate CYP1A1 enzyme activity in an Aryl hydrocarbon receptor-dependent manner^48^, and thereby promote bilirubin excretion. Thus, it seems possible that this mechanism improves bilirubin excretion (and thus lower bilirubin levels compared to DIO alone) in our DIO mice treated with exogenous bilirubin. Further studies beyond the scope of the current manuscript will be needed to test this hypothesis.

In this study, we found that short-term bilirubin treatment was associated with decreased total cholesterol and increased PPARγ and adipokines. These findings provide mechanistic evidence that bilirubin or altered bilirubin metabolism (e.g., partial UGT1A1 inhibitors^49^) may be useful as a therapeutic approach to reduce obesity and improve insulin resistance and glucose tolerance.

## Methods

### Animals

Male C57BL/6 mice (6 weeks old) were purchased from the Institute of Genetics and Developmental Biology, Chinese Academy of Sciences (Beijing, China). Mice were fed either a high fat diet (Table 1) or standard CHOW-fat diet (10% of calories from fat) for 24 weeks before treatment. The Animal Care Committee at the Qingdao Agricultural University approved all animal experiments. The methods were carried out in accordance with the approved guidelines.

### Bilirubin preparation and administration

Bilirubin (Frontier Scientific, Logan, UT) was dissolved in 0.1 N NaOH and the pH was adjusted to 7.4 using HCl. Bilirubin was administered intraperitoneally 20 μmol/kg (11.7 mg/kg) twice per day for 14 days. Control DIO or CHOW mice received vehicle on the same injection schedule.

### Mouse monitoring after treatment

Non-fasting serum blood glucose levels and body weights of mice were measured daily at 9:00 am. A drop of whole blood (about 5–10 μl) was collected from a minimal tail incision from end of the mouse’s tail and analyzed using a Sannuo glucometer (San Nuo Inc., Changsha, China). Physical activities of mice (including behavior, food intake, drinking, and licking) were observed on a daily basis as described^35^. Food intake (excluding spillage) was measured during a 24 h period at 7 days post-treatment, and weekly for 7 weeks after bilirubin treatment.

### Intraperitoneal glucose tolerance (IPGTT) and insulin tolerance test (ITT)

For IPGTT, mice were fasted overnight and then injected with 2 g/kg of glucose solution (i.p.). For ITT, mice were fasted for 5 hours and then injected with 0.75 U/kg insulin (i.p.; Eli Lili, Indianapolis, IN). Serum blood glucose levels were measured at 0, 15, 30, 60, 90, and 120 min after glucose or insulin injection.

### Tissue harvesting, serum preparation, and blood biochemistry

At the end of each experiment, mice were anesthetized and blood was collected by retroorbital bleeding into a heparinized tube. Plasma was obtained by centrifugation and stored at -80 °C for further analysis. Mice were then euthanized and livers and epididymal fat tissues were separated, weighed, and snap frozen in liquid nitrogen for later analysis. Serum TC, TG, HDL, LDL, and bilirubin levels were measured using specific reagent kits (Sigma Aldrich, Saint Louis, MO). Leptin, insulin, and adiponectin levels were measured by ELISA (R&D Systems, Minneapolis, MN).

### Real-Time (RT)-PCR analysis

Expression of the insulin receptor, leptin receptor, apolipoprotein A-IV (Apoa4), and SREBP-1 in livers were quantified by RT-PCR analysis as described previously^50^. β actin expression was quantified by RT-PCR in each sample and used as an endogenous control. Real time RT-PCR primers were purchased from Life Technologies (Invitrogen Trading Co., Ltd., Shanghai, China).

### Western blot analysis

Protein content of whole cell lysates were quantified by BCA assay, and 30 μg protein from each sample was separated on a 4–12% Bis-Tris gel (Beyotime Institute of Biotechnology, Haimen, China). Proteins were transferred to a Hybond-P membrane (GE healthcare, Piscataway, NJ), blocked with 5% non-fat milk, and incubated with the following primary antibodies: PPARγ, C/EBPα (Abcam, USA), and GAPDH (Santa Cruz Biotechnology Inc., USA), overnight at 4 °C. Blots were then probed with horseradish peroxidase labeled secondary antibodies (Sangon Biotech Ltd., Shanghai, China) 45 minutes at room temperature and visualized by an ECL detection kit (Amersham Pharmacia Biotech, Little Chalfont, UK). The intensity of each signal was determined using Image J software (NIH).

### Hematoxylin and eosin (H &E) staining

Immunohistochemistry of mouse liver and epididymal fat were performed as described^35^. Briefly, liver or adipose tissue sections were fixed in 10% buffered formalin overnight, embedded in paraffin, and sectioned. The slides were immersed in filtered hematoxylin for 6 min, rinsed with water, and stained with Eosin for another 1–2 min. Sections were rinsed with water and dehydrated in ascending alcohol solutions, cleared with xylene, and mounted with a cover slip onto a labeled glass slide. Slides were examined under a transflective dual-use Olympus BX51 microscope (Olympus, Tokyo, Japan), and images were captured using an Olympus DP72 digital camera. Vacuole area in total liver tissue area was determined in liver by the Image-Pro Plus analysis software. Diameters of individual fat cells (n = 75 in each group) were measured using CellSens Standard software (Olympus).

### Statistical analysis

Data are expressed as mean ± SD. Differences between two groups were compared for statistical significance using unpaired Student’s *t* tests with Bonferroni correction. Differences was considered significant when p < 0.05.

## Additional Information

**How to cite this article**: Liu, J. *et al*. Bilirubin Increases Insulin Sensitivity by Regulating Cholesterol Metabolism, Adipokines and PPARγ Levels. *Sci. Rep.* **5**, 9886; doi: 10.1038/srep09886 (2015).

## Figures and Tables

**Table 1:** Composition of the high fat diet.

**Ingredients**	**Percentage (%)**
Corn starch	27
Bran	19
Rice	16
Soybean cake	16
Fish powder	13
Calcium powder	3
Bone powder	3
Yeast extract	2.3
Salt	0.5
Vitamin mix	0.1
Mineral mix	0.1
