# Corrigendum: Bilirubin Increases Insulin Sensitivity by Regulating Cholesterol Metabolism, Adipokines and PPARγ Levels

**DOI:** 10.1038/srep19170

**Published:** 2016-01-20

**Authors:** Jinfeng Liu, Huansheng Dong, Yong Zhang, Mingjun Cao, Lili Song, Qingjie Pan, Andrew Bulmer, David B. Adams, Xiao Dong, Hongjun Wang

Scientific Reports 5: Article number: 9886; 10.1038/srep09886 published online: 05282015; updated: 01202016.

This Article contains an error in Affiliation 1. The correct affiliation is listed below:

College of Animal Science and Veterinary Medicine, Qingdao Agricultural University, Qingdao, P.R. China

